# Evaluation of Experiences With Ecological Momentary Assessment Among Women With Metastatic Breast Cancer: Qualitative Study

**DOI:** 10.2196/80467

**Published:** 2026-03-03

**Authors:** Emily P Taylor, Brenna Mossman, Mikaela A Velazquez-Sosa, Zaynab Rashid, Natalie Kloster, Maureen Schwarz, Hannah Kang, Jennifer D Rodriguez, Elizabeth L Addington, Judith Tedlie Moskowitz, Lesley Glenn, Shontè Drakeford, Roxana Guerra, Claudine Isaacs, Ami Chitalia, Christopher Gallagher, Deena Graham, Suzanne C O'Neill, Claire C Conley

**Affiliations:** 1 Lombardi Comprehensive Cancer Center, Georgetown University, 2115 Wisconsin Ave NW, Suite 300, Washington, DC, 20007, United States, +1 202-687-5086; 2 Department of Medical Social Sciences, Feinberg School of Medicine, Northwestern University, Chicago, IL, United States; 3 Patient Author, Project Life MBC, Calabasas, CA, United States; 4 Patient Author, Upper Marlboro, MD, United States; 5 Patient Author, Washington, DC, United States; 6 MedStar Washington Hospital Center, Washington, DC, United States; 7 John Theurer Cancer Center, Hackensack Meridian Health, Hackensack, NJ, United States

**Keywords:** quality of life, metastatic cancer, breast cancer, ecological momentary assessment, ecological momentary intervention, feasibility, acceptability, qualitative data

## Abstract

**Background:**

Patients with metastatic breast cancer (MBC) experience significant quality-of-life decrements, but there are few supportive care interventions specifically designed for this group that significantly improve quality of life. Ecological momentary assessment (EMA) and related ecological momentary interventions (EMIs) may be particularly beneficial for patients with MBC. However, no studies have previously examined the use of EMIs in the context of metastatic cancer.

**Objective:**

The purpose of this study was to qualitatively assess experiences with EMA and preferences for intervention content and mode among patients living with MBC, with an emphasis on EMIs.

**Methods:**

Women with MBC (n=29) were recruited from a longitudinal, observational study of quality of life using an EMA design. In-depth qualitative interviews assessed participants’ perspectives on the EMA design, including its feasibility, acceptability, and relevance to participants’ MBC experiences. Guided by participants’ EMA data, the interviews also examined the impact of self-monitoring and possible interventions when patients reported high symptom burden and/or low quality of life. Interviews were audio-recorded, transcribed verbatim, and analyzed using open coding, axial coding, and selective coding. The codebook was developed by reviewing a randomly selected subset of transcripts (n=5) and creating inductive, data-driven codes using the raw data, which were deductively organized into overarching themes.

**Results:**

Participants were mainly White (17/29, 59%), heterosexual (23/29, 79%), currently working (17/29, 59%), and held at least a bachelor’s degree (17/29, 59%). Participants had been living with MBC for a median of 2.5 years (range 0.2‐16.6 years). Most were diagnosed with hormone receptor-positive (23/29, 79%) and HER2-negative (21/29, 72%) breast cancer. Qualitative analysis identified 5 major themes. The participants reflected on their reasons for enrolling in the study, including interest in study activities and giving back to the MBC community (theme 1: participant engagement). Most participants found the EMA format easy to complete, and they provided recommendations for improving the design of future studies, including altering the timing and frequency of questionnaires (theme 2: feedback on study design). While less common, some participants discussed how engaging with the EMA protocol altered their experiences or behaviors (theme 3: impact of self-monitoring). Participants also discussed their reactions to seeing their EMA data, including their mixed thoughts on real-time data sharing (theme 4: responses to data). Finally, participants suggested programs and resources to improve their overall quality of life, reflecting on their interest in real-time interventions and peer-to-peer support (theme 5: recommendations for future interventions).

**Conclusions:**

Patients with MBC are willing to use EMA methodologies for data collection on quality of life and are open to EMIs with varying content and formats. Given variations in daily functioning between and within patients, a just-in-time adaptive intervention framework may be well suited for this population.

## Introduction

Metastatic breast cancer (MBC) is breast cancer that has spread to other parts of the body; it is typically considered to be treatable but incurable [1]. Approximately 5%‐10% of breast cancers are metastatic at diagnosis, and 20%‐30% of early-stage breast cancers will progress to MBC [2-4]. In the past decade, novel treatment approaches have resulted in significantly increased survival times following MBC diagnosis [5-10]. For this reason, the MBC population is anticipated to increase in the coming years; by 2030, an estimated 246,000 people in the United States will be living with MBC (a 59% increase from 2017) [11,12].

MBC symptoms, treatment side effects, and their impact on daily functioning influence quality of life (QOL) in this population [13,14]. Given the incurable nature of the disease, QOL is a priority for patients with MBC [15-17]. Interventions that improve QOL for people with MBC are vital; not only is QOL an important outcome in and of itself, but improvements in QOL may also enable patients to adhere to life-sustaining therapies [18].

However, there are few supportive care interventions that significantly improve QOL for people with MBC. A 2023 systematic review identified 11 studies testing supportive care interventions specifically designed to improve QOL among individuals living with MBC [19]—only 3 demonstrated QOL improvements. More recent trials have found that health-related QOL in patients with MBC significantly increased 6 months after the onset of a supervised exercise program [20]. Two small pilot trials, one examining the impact of brief psychotherapy [21] and another evaluating a whole-food, plant-based diet [22], demonstrated effects in some QOL domains, but not others. Thus, further research is needed to develop, test, and optimize interventions targeting QOL for patients with MBC.

Ecological momentary interventions (EMIs) may be particularly beneficial to help ameliorate QOL in patients with MBC. EMIs deliver dynamic, individually tailored treatments to patients in real time and in natural settings [23,24], using ecological momentary assessment (EMA) methodology (ie, repeated, in situ reporting) to identify optimal times to provide support. This type of adaptive intervention approach has several potential benefits over traditional “one-size-fits-all” interventions: maintaining participant engagement, sustaining continued behavior change for longer durations, and ultimately achieving greater intervention effects [25]. Given the substantial variability in QOL trajectories among patients with metastatic cancer generally [26]—and the widely acknowledged heterogeneity of the disease and treatment response in MBC specifically [27-29]—EMIs could be ideally suited to meet the needs of patients with MBC with the most QOL decrements without overburdening those who are currently stable. However, EMIs have rarely been used in cancer survivorship research [30]. To our knowledge, there are no studies examining EMIs in the context of metastatic cancer, and further information is therefore needed to inform their use within this population.

The purpose of this study was to qualitatively assess preferences for intervention content and mode among patients living with MBC, with an emphasis on EMIs. Participants were recruited from a longitudinal, observational study of QOL using an EMA design [31,32]. In-depth qualitative interviews assessed participants’ perspectives on the EMA design, including its feasibility, acceptability, and relevance to participants’ MBC experiences. As symptom monitoring can improve symptom control for patients with cancer [33-35], the interviews also examined the impact of self-monitoring. Finally, guided by participants’ EMA data, interviews solicited suggestions for possible interventions when patients reported high symptom burden and/or low QOL.

## Methods

### Procedures and Participants

This study used qualitative exit interview data from a longitudinal study of QOL among patients with MBC. Data collection and analyses were guided by a pragmatist philosophical orientation and align with COREQ (Consolidated Criteria for Reporting Qualitative Research) [36].

Between January 2023 and April 2024, participants were recruited from 5 sites within the Georgetown Lombardi Comprehensive Cancer Center Network (in Washington DC, Maryland, and New Jersey). Eligible participants were assigned female at birth, aged 18 years and older, diagnosed with MBC, able to speak English or Spanish, and had a working telephone capable of receiving SMS text messages and accessing the internet. Study team members screened the electronic medical record to identify potentially eligible patients, obtained approval to approach participants from their treating oncologist, approached potentially eligible patients via telephone, and confirmed patient eligibility. Eligible, interested patients provided written informed consent via electronic signature on web-based consent forms. Following informed consent, participants completed a brief baseline survey and a 4-week EMA protocol. Participants completed 3 EMA prompts per day, 1 day per week (randomly assigned each week), for 4 consecutive weeks; thus, participants completed up to 12 EMA surveys. This “measurement burst” design incorporates short periods of intensive, recurrent assessment that are repeated longitudinally and spaced out over longer intervals. This design reduces participant burden while maintaining density of assessments [37,38]. EMA prompts consisted of a text message with a link to a REDCap (Research Electronic Data Capture) survey (Multimedia Appendix 1). Each survey assessed global QOL, symptom severity (depression, anxiety, pain, fatigue, slowed cognitive functioning, appetite loss, nausea, gastrointestinal [GI] distress, and decreased libido), and positive experiences (social connectedness, peace, and joy). Participants had a 3-hour window to complete each EMA. Thus, EMA surveys enabled assessment of change in each domain over the course of the day and over the course of 1 month (the approximate length of a treatment cycle for a patient with MBC).

Upon completion of EMA data collection, participants were queried regarding their interest in and willingness to participate in an exit interview. Initially, we planned to offer the exit interview to all study participants. However, in the first 3 months of data collection, a relatively large number of participants were interested in participating in the exit interview (20/24, 83% of participants enrolled in the first 3 months). As it would not be feasible to conduct interviews and analyze qualitative data from more than 80% of study participants (target n=120), we elected to change our approach and randomly sample 15% of participants who were interested in the exit interview. Sampling for the interview was stratified by recruitment site, such that 15% of participants from each site who were interested in the exit interview were invited to participate. We engaged in constant comparative analysis [39] and conducted interviews until theoretical saturation was reached [40].

Three female members of the study team with prior training and experience in qualitative interviewing (CCC, MAVS, and JDR) conducted semistructured qualitative interviews in English or Spanish with selected participants. The interviewers did not have a relationship with the interviewees prior to their participation in the study but had sometimes interacted with the participants during the EMA phase. Interviewers reintroduced themselves and reminded participants of the rationale for the study at the start of the interview. The interviewers had foundational knowledge of MBC but not firsthand experience. They also tended to be younger than the interview participants. These characteristics may introduce bias by affecting the interviewer’s application of probing questions. Potential bias was managed through postinterview debriefing with colleagues.

**Figure 1.**
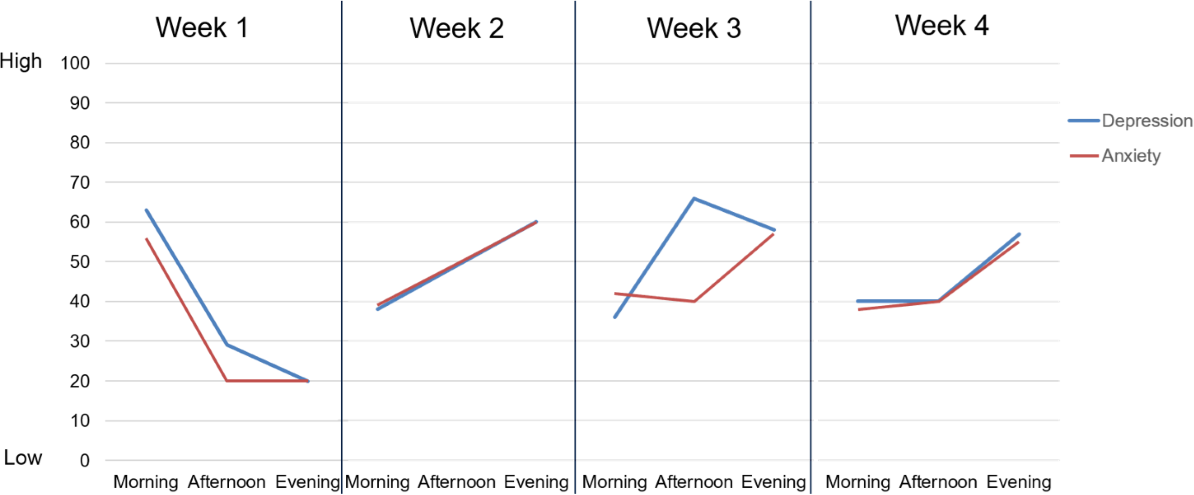
Example ecological momentary assessment (EMA) data display figure, showing one participant’s responses to EMA questions on anxiety and depression across 12 time points (morning, afternoon, and evening; 1 day per week for 4 consecutive weeks). The vertical axis represents the visual analog scale ranging from 0 to 100 and the horizontal axis represents the EMA time point. The blue lines indicate depression scores and the red lines indicate anxiety scores.

The interview guide (Multimedia Appendix 2) was developed by the study team in collaboration with patient advocates with MBC. We first identified broad topic areas of interest that included both feedback on their experiences in this study and the perceived acceptability of future research projects, including EMIs. Once the topic areas for the interview were identified, the group brainstormed a wide variety of probing questions that could be used to elicit additional input from participants. Thus, the resulting interviews began with open-ended questions about participants’ experiences with the EMA protocol and symptom self-monitoring. After the open-ended questions, participants were shown their EMA data in graphical format (see Figure 1 for an example). Specifically, participants were shown a series of line graphs representing their responses to the EMA surveys over the 4 weeks in the study. Participants viewed graphs representing overall QOL, mental health (anxiety and depression), physical health (pain, fatigue, nausea, and GI distress), thinking and feeling (slowed cognitive functioning, appetite loss, and decreased libido), and positive experiences (social connectedness, peace, and joy). The EMA data display figures were developed by the study team and iteratively revised based on feedback from patient advocates with MBC to ensure clarity prior to their use in the interviews. For each graph, the interviewer oriented the participant to the horizontal axis, vertical axis, and legend. The interviewer also answered any questions about the data visualization. Participants were then asked about their reactions to their data, how they might change their behavior in response to these data, and their preferences for data-guided symptom management interventions.

Interviews took place via telephone or web-based video platform, based on participant preference. When interviews took place via telephone, participants were mailed or emailed the EMA data display figures and were instructed to refer to them during the telephone call. Interviews lasted 44 minutes on average (range 26‐76 minutes), were audio-recorded, transcribed verbatim, and translated to English (if necessary). The interviewer took field notes during the interviews. As participants did not consent to be recontacted, we did not conduct repeat interviews, return transcripts to participants for review, or have participants provide feedback on the findings.

### Data Analysis

We used a combined deductive and inductive qualitative data analysis approach [41,42]. First, 3 members of the study team (CCC, MAVS, and EPT) reviewed a randomly selected subset of transcripts (n=5) and collaboratively developed a detailed codebook consisting of data-driven codes from the raw data [43]. These codes were then organized based around core concepts of feasibility and acceptability as reflected by the topic areas in the interview guide. Thus, the data-driven codes represented inductive subthemes that were deductively organized into themes based on our belief that certain core concepts exist in the data [44,45]. Following the development of the codebook, all transcripts were independently coded by 2 of 6 raters (MAVS, ZR, NK, EPT, MS, and HK). Coding disagreements were resolved through discussion with a third rater (CCC) until consensus was reached. Coding was conducted using Dedoose.

### Ethical Considerations

All procedures were reviewed and approved by the Institutional Review Board at Georgetown University (institutional review board number 00005081). All participants provided written informed consent via electronic signature on web-based consent forms and confirmed ongoing consent verbally at the time of the qualitative interview. Informed consent included consent for the use of quotes in research publications. Qualitative data are deidentified to protect participant confidentiality. Participants received US $10 for completing the baseline survey, up to US $25 for completing EMA surveys, and US $20 upon interview completion. Thus, participants could earn up to US $55 for completing all study activities. Participant incentives were provided as a gift card.

## Results

### Participant Demographics

**Figure 2.**
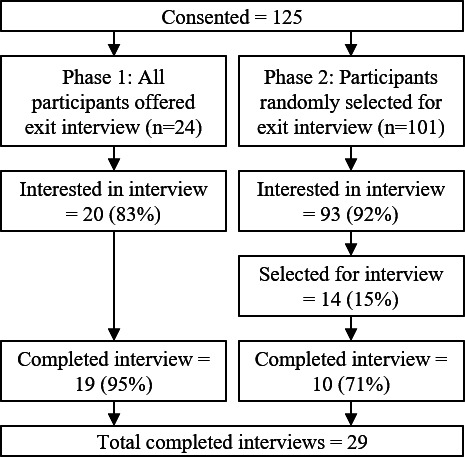
Study flow diagram.

Of the 125 eligible patients who consented, 113 out of 125 (90%) expressed interest in the exit interview, 34 out of 113 (30%) were invited to participate in the interview, and 29 out of 34 (85%) completed the interview (Figure 2).

See Table 1 for a full description of the interview participants. The median participant age was 57 years. The majority were non-Hispanic White (17/29, 59%), currently working (17/29, 59%), and held a bachelor’s degree or greater (17/29, 59%).

Participants had been living with MBC for a median of 2.5 years (range 0.2‐16.6). Most participants were diagnosed with hormone receptor positive (23/29, 79%) and HER2 negative (21/29, 72%) breast cancer. The most common treatments received were targeted therapy (20/29, 69%) and hormone therapy (16/29, 55%); chemotherapy (7/29, 24%), immunotherapy (5/29, 17%), and radiation therapy (1/29, 3%) were less common. Most participants had an Eastern Cooperative Oncology Group performance status rating of 0 (13/29, 45%) or 1 (11/29, 38%), indicating no performance restrictions and restrictions in strenuous physical activity, respectively.

**Table 1.** Participant demographic and clinical characteristics (N=29).

Characteristics	Values
Demographic characteristics (self-reported)	
Age (years)	
Mean (SD)	54.8 (11.5)
Median (range)	57 (33-74)
Race and ethnicity^a^, n (%)	
Asian	1 (3)
Black/African American	8 (28)
Hispanic/Latina	3 (10)
Native Hawaiian/Pacific Islander	1 (3)
White	17 (59)
Preferred language, n (%)	
English	27 (93)
Spanish	2 (7)
Sexual orientation, n (%)	
Heterosexual or straight	23 (79)
Lesbian	3 (10)
Bisexual	1 (3)
Missing/prefer not to answer	2 (7)
Partner status, n (%)	
Partnered	18 (62)
Not partnered	10 (35)
Missing/prefer not to answer	1 (3)
Education, n (%)	
Less than bachelor’s degree	12 (41)
Bachelor’s degree or greater	17 (59)
Employment status, n (%)	
Currently working	17 (59)
Not working	10 (35)
Missing/prefer not to answer	2 (7)
Annual household income, n (%)	
<US $100,000	18 (62)
≥US $100,000	7 (24)
Missing/prefer not to answer	4 (14)
Health insurance type^a^, n (%)	
Private	23 (79)
Public (eg, Medicare and Medicaid)	9 (31)
Missing/prefer not to answer	1 (3)
Clinical characteristics (abstracted from EMR)^b^	
Years since MBC^c^ diagnosis	
Mean (SD)	4.3 (4.1)
Median (range)	2.5 (0.2-16.6)
Hormone receptor status, n (%)	
Positive	23 (79)
Negative	6 (21)
HER2 status, n (%)	
Positive	8 (28)
Negative	21 (72)
Treatment(s) during study^a^, n (%)	
Chemotherapy	7 (24)
Radiation therapy	1 (3)
Immunotherapy	5 (17)
Targeted therapy	20 (69)
Hormone therapy	16 (55)
ECOG^d^ performance status, n (%)	
0	13 (45)
1	11 (38)
2	1 (3)
Missing	4 (14)
Study characteristics	
Study site, n (%)	
MedStar Georgetown University Hospital	10 (34)
MedStar Washington Hospital Center	9 (31)
MedStar Good Samaritan Hospital	2 (7)
MedStar Franklin Square Medical Center	4 (14)
Hackensack Meridian Health/John Theurer Cancer Center	4 (14)
Number of EMA^e^ surveys completed	
Mean (SD)	10.7 (1.6)
Median (range)	11 (6-12)

a As participants could select more than 1 response for race and ethnicity and health insurance type, and could be receiving multiple treatments simultaneously, counts for these categories sum to more than the sample total.

b EMR: electronic medical record.

c MBC: metastatic breast cancer.

d ECOG: Eastern Cooperative Oncology Group.

e EMA: ecological momentary assessment.

### Themes

Qualitative analysis identified 5 major themes. Table 2 provides theoretically driven themes and the component subthemes derived from the raw qualitative data. Textbox 1 provides exemplar quotes from participants for themes 1‐4. Exemplar quotes for theme 5 are included in Textbox 2.

**Table 2.** Theoretically driven themes and data-driven subthemes.

Themes	Subthemes
Participant engagement	Motivation for participation or altruism Feeling seen Interest in study activities
Feedback on study design	Demystifying or clarity about study Flexibility and accessibility Frequency of assessments Timing of assessments Response options Technology Relevance of the questions
Impact of self-monitoring	Emotional reactions to study activities or procedures Heightened awareness Behavior changes
Responses to data	Reflecting on the data Positive experiences Reaction to the idea of real-time data sharing
Recommendations for future interventions	Content Timing Format Audience Peer support

Textbox 1. **Representative quotes illustrating themes 1-4.**

**Participant engagement**

- “I like being part of a study in that I think it’s good to get a wide range of how people experience cancer and everything like that, so.” [Participant 16, 9 years post-MBC diagnosis, completed 12 EMA surveys]
- “These things are good experiences for people. I mean, you guys get insight and I get to think about how I actually am feeling at those times.” [Participant 19, 1 year post-MBC diagnosis, completed 8 EMA surveys]
- “Well, I really respect my oncologist. So the fact that she had passed me along for it made me want to do it. And then I think looking at quality of life issues for people with metastatic breast cancer, I felt a connection to the study.” [Participant 20, 9 years post-MBC diagnosis, completed 12 EMA surveys]

**Feedback on study design**

- “It was very easy to participate. And I haven’t given it much thought because I would just do it. And it wasn’t difficult in any way.” [Participant 12, 5 years post-MBC diagnosis, completed 10 EMA surveys]
- “Instead of looking at overall quality of life, we are being asked to look at it in snippets of time. And I think you can get a skewed answer that way. It really depends on how the participant is looking at the question.” [Participant 27, 3 years post-MBC diagnosis, completed 7 EMA surveys]
- “I think the three times in one day, I don’t know if that was really needed. I would rather have done more [days] because it would key in more on if you’re having a good day or a bad day. Because I can remember some of my bad days. I thought, ‘Oh, I wish I had the questions.’ [My answers] would be so different.” [Participant 17, 2 years post-MBC diagnosis, completed 12 EMA surveys]
- “I appreciated the fact that I could take the survey on my phone, so no matter where I was, I was able to access it. That was really good.” [Participant 4, 10 years post-MBC diagnosis, completed 10 EMA surveys]
- “I wish that I could have done it over email because sometimes it’s hard for me to get to my phone. But I liked it when you guys texted me the night before so I knew that I would have to check my phone a few times a day.” [Participant 14, 5 months post-MBC diagnosis, completed 12 EMA surveys]
- “I thought the questions were very good. They touched on a lot of different parts of your life that you really wouldn’t normally think about.” [Participant 17, 2 years post-MBC diagnosis, completed 12 EMA surveys]
- “Some of [the questions] didn’t apply to me. I’m not sexually active, so I didn’t know how to gauge that.” [Participant 4, 10 years post-MBC diagnosis, completed 10 EMA surveys]

**Impact of self-monitoring**

- “It did make me more cognizant and mindful. It made me think, ‘Is there something I can do for myself?’ Having those little pauses was actually nice. And at times it made me kind of grateful in that moment that not everything was challenging.” [Participant 24, 3 years post-MBC diagnosis, completed 12 EMA surveys]
- “It just made me think about what I’m going through because sometimes you just go through the day, and you don’t pay much attention to what you’re feeling. And this really made me think more about what’s happening, and if it wasn’t good, how can I change it or how could I feel different...It makes you ask yourself, ‘How am I taking care of myself? Am I doing a good job or am I not?’ Because that’s important.” [Participant 17, 2 years post-MBC diagnosis, completed 12 EMA surveys]
- “I don’t think I changed [my behavior]. I think I became more mindful of things...I can’t think of an instance where I was like, ‘Oh, let me go do more self-care.’ I think it just made me mindful of what I was doing.” [Participant 6, 2 years post-MBC diagnosis, completed 11 EMA surveys]
- “I was more aware of my behavior, aware of myself like I was more conscious of how I was feeling on what I was doing. For instance, when I tell myself, ‘I don't feel like exercising today,’ or, ‘I’m too tired to go downstairs on the treadmill,’ but then I have to tell myself, ‘You’ll feel better. You need it.’ So just things like that. It just made me more aware...It’s like you’re holding yourself accountable.” [Participant 18, 3 years post-MBC diagnosis, completed 12 EMA surveys]

**Responses to data**

- “I was a little hesitant [to look at my data] because I kind of was expecting this...Unfortunately, I think this is probably pretty accurate and describes my highs and lows and the stress and overwhelm that I had been feeling...But I guess the anxiety led to more of the overwhelm, feeling alone, challenging, more depressive thoughts and feelings. So unfortunately, this is probably really, really accurate.” [Participant 24, 3 years post-MBC diagnosis, completed 12 EMA surveys]
- “Overall, I try to stay very positive, but when you get granular with specific feelings and experiences and whatnot, yeah, it probably doesn’t equate to the quality of life being so high...As I look at [my data], I’m like, ‘Well, how the heck do you get that [high] quality of life then?’ Because it just doesn’t seem like anything should—none of these average to the quality of life that I thought I had. But at the end of the day—so food didn’t taste good to me today and whatever, but is it really affecting my quality of life? I’m just happy to be here. Just very lucky. Very, very thankful.” [Participant 25, 1 year post-MBC diagnosis, completed 11 EMA surveys]

Textbox 2. **Representative quotes illustrating theme 5 and related subthemes.**

**Content**

- “[I would like] suggestions that I could implement myself without needing assistance from someone else. Definitely things that would have the quickest effect.” [Participant 3, 2 years post-MBC diagnosis, completed 11 EMA surveys]
- “You can send more tips of that meditation, of just that deep breathing...I think deep breathing makes a big difference. Even if you don’t know how to meditate, to teach someone how to do the deep breathing, to help them through those anxiety attacks will help.” [Participant 18, 3 years post-MBC diagnosis, completed 12 EMA surveys]
- “Maybe you can send ways to calm yourself down in the moment. Like take a step back, breathe in, breathe out, put your arms up, whatever. Things like that. Or if you need to punch a pillow or something. So it might be more intense, but it has ideas that are safe for you to get it out. Go in the bathroom and let it out. Find yourself a place or just take a pause in whatever you’re doing, just different things like that.” [Participant 7, 2 months post-MBC diagnosis, completed 10 EMA surveys]
- “Some kind of question to prompt thought. Like, ‘When have you last eaten, or have you had some water recently? Can you get a few minutes of fresh air or talk to somebody or think of an affirmation or write something down?’ Any of those kinds of things, I guess. Just something to be more mindful and practical.” [Participant 24, 3 years post-MBC diagnosis, completed 12 EMA surveys]
- “I guess for nausea, to remind you of all the interventions and to make sure that you're on top of it. It’s so important with nausea to stay ahead of it. Same with pain management or GI distress, really, too.” [Participant 20, 9 years post-MBC diagnosis, completed 12 EMA surveys]
- “I know that there are studies about how getting out in nature lifts your spirits and exercise and that sort of thing. So maybe encouragement to do that...Or kind of preemptive solutions that might hit a larger number of people. Like, ‘Oh, look, they reminded me this week that it’s spring so your allergies might be worse because your immune system is compromised so do this. Exercise inside instead of jogging outside.’” [Participant 15, 8 years post-MBC diagnosis, completed 12 EMA surveys]
- “My doctors tell me that they have resources out there that will help a person. So if I was feeling depressed, I would probably want to be guided somewhere where I can talk to a mental health person, or talk to someone about a type of medication, or support groups or stuff like that. Maybe you can tell me, ‘Okay. If you use this link, that can take you to where you need to get the help that you think you might need.’ Because there’s so much information out there. Sometimes you can’t concentrate.” [Participant 1, 1 year post-MBC diagnosis, completed 10 EMA surveys]

**Timing**

- “If you rank between this and this [on the survey], maybe they could send them to you just as a, ‘Hey, you’re not alone. We appreciate your feedback. And while it may not totally resolve your matter, here are some suggestions.’ Right after you finish that survey for that time period or something because some people may not know what to do, just suggestions.” [Participant 7, 2 months post-MBC diagnosis, completed 10 EMA surveys]
- “I feel like [it would suggest] things that I already do, which are listen to music and meditate and pray. But just, I guess, suggestions to help in the moment.” [Participant 13, 1 year post-MBC diagnosis, completed 12 EMA surveys]
- “I think a prompt would be nice. [Something] that’s nudging you to go and look at it, but if you don’t want to, you can opt out. I think the idea of having a nudge to go look at something would be nice based off reporting something as being lower and then that kind of correlating with the app prompting.” [Participant 23, 2 years post-MBC diagnosis, completed 12 EMA surveys]
- “I think it depends. That week when I was having a really tough time, I might not have been able to take another piece of advice that day. So maybe it’s a follow-up on the next day...Just based on that one particular day, I might have just been like, ‘No, you have no idea. Nothing is going to help me today.’ But maybe the next day? Like, ‘Okay. The next time you find yourself having that kind of thinking or this, why don’t you maybe try one of these suggestions? Keep this in your pocket.’” [Participant 25, 1 year post-MBC diagnosis, completed 11 EMA surveys]

**Format**

- “If there’s a website you can go to and it has different topics—‘I’m feeling a lot of fatigue today. What are some of the things I can do?’ You click on a button and see, ‘Go outside and take a brisk walk.’ Yes, practical application is what I’m looking for.” [Participant 3, 2 years post-MBC diagnosis, completed 11 EMA surveys]
- “Nothing too lengthy. For me, my attention span isn't all that great...I don’t mind reading but nothing that’s going to take more than like 15 minutes of my time. I just get distracted and impatient.” [Participant 13, 1 year post-MBC diagnosis, completed 12 EMA surveys]
- “Well, I mean, people could choose. There’s websites you can go on to get free [meditation videos] and there’s also websites that have some wonderful healing music on too.” [Participant 9, 4 years post-MBC diagnosis, completed 9 EMA surveys]

**Audience**

- “It’s so complicated because there are people who suffer from very difficult symptoms, who sometimes don’t feel like going for a walk. But if the family supports them, encouraging them to take a break, ‘Let’s just walk for 15, 20, 30 minutes’ that can help a lot. Because there are times when those chemotherapies are so strong, it’s really difficult to tell a person, ‘Go for a walk.’ So, if the family supports them, or friends accompany them, maybe the person will be a little more encouraged.” [Participant 29, 10 years post-MBC diagnosis, completed 12 EMA surveys]
- “My husband is not one of those people that likes to talk or even believes in therapy, but he’s a people person. So he’s related to some of his coworkers and things like that that have had wives or spouses that have had cancer. I know that that’s helped him. I just wish that there was somebody for my kids and my husband to talk to.” [Participant 7, 2 months post-MBC diagnosis, completed 10 EMA surveys]

**Peer support**

- “What would help? Sometimes talking to somebody...If I’m really down in the dumps, I would not call my friends, though, because they don’t understand. But I would call another cancer patient. That’s how I would handle it for me. I think that would help me, because I know who I can call.” [Participant 8, 8 years post-MBC diagnosis, completed 11 EMA surveys]
- “I think reaching out just by phone to talk to somebody, just having a phone buddy or something like that. I think having someone to talk to would be important. Not so much to complain, but to let them know what’s going on. And then maybe have the person talk about good self-care.” [Participant 9, 4 years post-MBC diagnosis, completed 9 EMA surveys]

#### Theme 1: Participant Engagement

 *Participants primarily enrolled in the study because they wanted to give back to the MBC community*. Participants cited an altruistic drive as motivation for joining the study, stating “it’s my turn to give back a little bit” [Participant 26, 9 years post-MBC diagnosis, completed 10 EMA surveys]. Other participants expressed a desire to join the study to provide representation for specific subpopulations within the MBC community. For example, one participant stated that they enrolled in the study for “the opportunity to give some data points for women of color” [Participant 22, 2 years post-MBC diagnosis, completed 11 EMA surveys]. Furthermore, participants appreciated the study’s focus on their QOL, which they felt was an understudied dimension of MBC: “This is an area where there could be more support and research and more information out there for people who are just so busy and dealing with [MBC] and living with [MBC]” [Participant 24, 3 years post-MBC diagnosis, completed 12 EMA surveys].

 *Participants also appreciated the low-burden nature of the study procedures**.*** The 1-month assessment time frame meant that they “didn’t have to make a big commitment” [Participant 26, 9 years post-MBC diagnosis, completed 10 EMA surveys], and the lack of financial restrictions to enroll encouraged participation because “it doesn’t cost anything” [Participant 7, 2 months post-MBC diagnosis, completed 10 EMA surveys].

#### Theme 2: Feedback on Study Design

*Most participants felt the EMA design was straightforward**.*** Almost all participants noted that the daily assessments were simple to complete, especially given the reminders provided the day prior. One participant reflected that “It was quick and straightforward, very, very user-friendly, and very easy for somebody who could be forgetful like me and just be dealing with a lot all at once. It didn’t feel like it was one more thing that I had to do, which was really nice” [Participant 24, 3 years post-MBC diagnosis, completed 12 EMA surveys].

*However, some participants reflected on a lack of clarity in study activities**.*** Several participants noted that they did not fully understand the study procedures until they were actively completing them. For example, one participant had thought that they would receive surveys daily, rather than 1 day each week. Other participants did not grasp the overarching goal of the study and/or how the design would answer the research questions. As one participant shared, “I guess I just didn’t really understand what [the researchers] were looking for” [Participant 21, 17 years post-MBC diagnosis, completed 11 EMA surveys].

*When asked about alternative study designs, participants suggested fewer assessments per day, with a larger response window, for multiple days in a row*. Some participants had trouble responding to surveys within 3 hours. One participant noted, “Sometimes, I found it difficult to keep up with actually answering the questions in a timely manner. Just regular everyday time management sometimes didn’t work out the way I needed it to” [Participant 3, 2 years post-MBC diagnosis, completed 11 EMA surveys]. Thus, allowing a longer response window could improve response rates. Generally, participants felt that the 3 surveys administered per day were more than sufficient to adequately capture their experiences that day; some noted that 1 or 2 surveys would have sufficed. However, participants frequently stated that completing surveys several days in a row would be a better reflection of their experiences. As one participant put it, “There were days that I wasn’t doing well, but those weren’t the days that the questions were. So in a way, I felt like it wasn’t accurate to how I was feeling all the time” [Participant 17, 2 years post-MBC diagnosis, completed 12 EMA surveys].

*Participants had mixed experiences with the technology used to deliver the study surveys*. Some participants enjoyed completing surveys on their cell phone, as this allowed them to do the survey in any location and did not interrupt their daily plans. Other participants would have preferred email surveys because it was sometimes difficult for them to get to the phone.

Another commonly cited technology issue was the use of sliders to capture symptom data, rather than a Likert-style response scale. Participants felt that the slider did not always reflect their experiences and that inadvertently moving the slider resulted in inaccuracies in their data. Numerical answers were preferred over sliders “because, with numbers, I could have done what I wanted to tell you with more precision” [Participant 10, 1 year post-MBC diagnosis, completed 11 EMA surveys].

Relatedly, many participants wished the study had involved more open-ended questions or room for comments. One participant noted:

*I thought it was a little too restrictive regarding the type of questions they were asking and my limited ability to be able to express what I was actually trying to think about, being forced to gauge high or low, where it would have helped me [is] if I could put in a little comment section. This is what I’m feeling. This is why I’m feeling it*.

[Participant 4, 10 years post-MBC diagnosis, completed 10 EMA surveys]

*Participants felt that most questions resonated with their MBC experiences, with the exception of one: libido*. Several participants noted that the questions resonated with their lived experiences with MBC, with the surveys serving as both a clarifying and reflective experience. For example, one participant reflected, “It was easy [to answer the questions] because I’m living it. So it’s not really something I have to think about” [Participant 19, 1 year post-MBC diagnosis, completed 8 EMA surveys].

The one exception to this pattern was the item assessing changes in libido. This item prompted many negative reactions; participants felt “uncomfortable” [Participant 7, 2 months post-MBC diagnosis, completed 10 EMA surveys] or “sad” [Participant 16, 9 years post-MBC diagnosis, completed 12 EMA surveys] and were “sick of being asked about [it]” [Participant 15, 8 years post-MBC diagnosis, completed 12 EMA surveys]. Confusion was another common reaction. Many participants did not realize that changes in libido can be a side effect of cancer treatment and were confused about why this would be included in a study of QOL in MBC. Others understood libido “as sex” [Participant 8, 8 years post-MBC diagnosis, completed 11 EMA surveys], which led to some confusion on the premise of the question and what was being asked, with the sense that it was not relevant for their personal situation (particularly for participants who were not partnered). Participants suggested that future studies assessing libido should include a “not applicable” response option.

#### Theme 3: Impact of Self-Monitoring

*Some participants described negative emotional responses to the study activities or procedures*. For example, one participant stated they felt “upset” answering questions about their QOL on “one of the days [they were not] really feeling good” [Participant 28, 3 years post-MBC diagnosis, completed 10 EMA surveys]. Another participant described their experience in detail:

*I mean, I’m not sure if upsetting is the right word, but it was kind of like, “I don’t want to go to that space right now.” Sometimes I was like, “I’m focused on something completely different, and that’s going to shift my perspective.” The survey itself doesn’t take very long to fill out, but yeah, your mind does kind of go in that space, and you start to think about that, which is distracting, potentially, to what your current day’s achievement outcomes and focus that you want it to be...when I finished the surveys or if it was a survey day, I don’t think I left feeling upset. But I think, yeah, a lighter version of distressing...*

[Participant 23, 2 years post-MBC diagnosis, completed 12 EMA surveys]

In other words, the survey prompt tended to interrupt participants’ usual routines and, in doing so, sometimes caused distraction or distress as they reflected on their symptoms and experiences.

*While participants generally expressed heightened awareness in response to the study assessments, the extent to which this resulted in behavior change varied.* Several participants expressed that completing the study assessments made them feel more self-aware, and they were appreciative of the opportunity to reflect on their feelings and experiences:

*It was amazing to actually capture my daily activities so I would be prepared to answer the questions. So I really was very focused on me and my feelings, which sometimes I will try to avoid and just stay busy. But this actually made me kind of reassess myself and see that I want to keep focusing on certain things, or just go on and enjoy life and do some of the things I wanted to do*.

[Participant 5, 2 years post-MBC diagnosis, completed 6 EMA surveys]

While this reflective experience prompted some to adopt certain behavior changes—such as exercise, yoga, or cooking a meal—many participants reported no explicit behavior change as a result of study participation. As one participant stated, “I thought the survey was very helpful, but it didn’t change me” [Participant 4, 10 years post-MBC diagnosis, completed 10 EMA surveys].

#### Theme 4: Responses to Data

*Participants appreciated the opportunity to see their data at the end of the study.* In reviewing their data, participants felt that they could more clearly see how it was going to be used to accomplish the goals of the study. As one participant noted:

*I really appreciate the fact that you are sharing all the information with me, and the opportunity to know what happened at the end. So that’s interesting for me because I never had that before, and I’ve been in a couple other studies.*

[Participant 28, 3 years post-MBC diagnosis, completed 10 EMA surveys]

*Many participants found their data interesting or surprising but individual reactions varied*. Some participants had positive responses when presented with data that they characterized as “good,” while others expressed sadness upon “seeing that [quality of life] was trending down” [Participant 16, 9 years post-MBC diagnosis, completed 12 EMA surveys]. While most participants felt that the graphs accurately reflected their weekly experiences, others commented on the inaccuracy of some data due to “user error” [Participant 1, 1 year post-MBC diagnosis, completed 10 EMA surveys]. This “user error” most frequently referred to inadvertently choosing the wrong response value on the slider (see theme 2).

Of particular note were participant reactions to their data on positive experiences, including joy, peace, and social connection. Some attributed low scores directly to their physical or mental health, or even felt that positive experiences were not possible for someone with cancer: “As my other friend with cancer and I have discussed, joy is not really much of a part of our experience. So I’m not surprised it never got over 50%” [Participant 15, 8 years post-MBC diagnosis, completed 12 EMA surveys]. However, many of the participants reported positive experiences *despite* difficulties with their physical or mental health:

*[My] physical health has declined. I have neuropathy so bad. It’s hard to hold things. My feet just dangle. I have no control over my feet. I don’t even get out of my pajamas, or some days I don’t even brush my teeth. But positive experiences, I do have some still. I still have my friends that call me and visit me sometimes.*

[Participant 8, 8 years post-MBC diagnosis, completed 11 EMA surveys]

*I have chronic neck pain. I have [cancerous] lesions top to bottom on every vertebra of my spine, and the worst being my cervical spine. And that’s where I had the surgery last year. So it’s healed. It’s strong. I mean, I was almost paralyzed last year, and I’m nowhere near that. I have a normal life, except I do have chronic pain in my neck sometimes...[but] my family and my kids bring me so much peace and joy*.

[Participant 13, 1 year post-MBC diagnosis, completed 12 EMA surveys]

*Reactions to the idea of real-time data sharing were mixed*. Interviewers asked participants how they would feel if their data were available in real time. Some participants felt that this would be beneficial:

*I think it would have been helpful. If I could see them in real time, then I would be aware of the things that I could change and make an effort to change them. Obviously, some stuff you can’t really change with pain and anxiety and stuff. That stuff is hard to change. But in terms of joy and peace, there are things you can do to try to change those*.

[Participant 3, 2 years post-MBC diagnosis, completed 11 EMA surveys]

Others felt that real-time data sharing, especially in moments of high depression or anxiety, would not be helpful. Many participants shared that they “don’t think [they] would have responded any differently” or changed their behaviors if they had received data in real time and preferred to “look at it all together in a month” to “get a better picture” [Participant 18, 3 years post-MBC diagnosis, completed 12 EMA surveys].

#### Theme 5: Recommendations for Future Interventions

*Real-time interventions were of interest to most, but not all, patients with MBC with various suggestions for content and format.* Participants were asked about their openness to real-time interventions in moments of high symptom burden. Many participants were open to receiving resources in real time. Suggested content for EMIs varied widely and included mindfulness, meditation, and breathing exercises; tips for emotion regulation; reminders to take medications for side effects such as pain, nausea, and GI distress; suggestions to engage in physical activity or spend time outside or in nature; affirmations and journaling; and prompts to connect with other people. Participants overall emphasized desire to see “practical application(s)” in real-time interventions to alleviate symptom burden [Participant 3, 2 years post-MBC diagnosis, completed 11 EMA surveys]. Some participants wanted real-time interventions that could “[be implemented] without the need for someone else” [Participant 3, 2 years post-MBC diagnosis, completed 11 EMA surveys]. These wishes for quick, pragmatic, and implementable interventions were consistent across participants who expressed interest in real-time interventions. In terms of the format of the intervention, participants overall expressed a preference for receiving resources via email or text message, preferably with a link to a website where they could access further information as needed. As one participant put it:

*If you rank between this and this [on the survey], maybe they could send them to you just as a, “Hey, you’re not alone. We appreciate your feedback. And while it may not totally resolve your matter, here are some suggestions.” Right after you finish that survey for that time period or something because some people may not know what to do, just suggestions.*

[Participant 7, 2 months post-MBC diagnosis, completed 10 EMA surveys]

Relatedly, one participant described how the frequency of real-time interventions could affect their adherence, sharing, “I like messages sporadically, something that draws my attention to it. It can’t be too habitual” [Participant 24, 3 years post-MBC diagnosis, completed 12 EMA surveys]. Less commonly, some participants suggested that a responsive, but delayed, intervention would be preferable. As one participant shared:

*That week when I was having a really tough time, I might not have been able to take another piece of advice that day...maybe the next day?*

[Participant 25, 1 year post-MBC diagnosis, completed 11 EMA surveys]

*Some participants saw potential benefits of EMIs for their friends and family*. While most participants felt that resources should be delivered directly to the person living with MBC, a few noted that their friends and family might also benefit from resources. For example, one participant expressed a desire for “somebody for my kids and my husband to talk to” [Participant 7, 2 months post-MBC diagnosis, completed 10 EMA surveys]. Another noted that engaging friends and family members might help bolster the overall effect of the intervention, stating “if the family supports them, or friends accompany them, maybe the person will be a little more encouraged” [Participant 29, 10 years post-MBC diagnosis, completed 12 EMA surveys].

*Many participants were interested in interventions that included engagement with other people with similar experiences.* Participants expressed a desire to connect with other patients with MBC, particularly in moments of high distress: “If I’m really down in the dumps, I would not call my friends, though, because they don’t understand. But I would call another cancer patient” [Participant 8, 8 years post-MBC diagnosis, completed 11 EMA surveys]. Some participants had been able to establish their own peer support networks but felt that providing support resources could help others. As one participant noted:

*I think that there are people who don’t know how to access those supports. So maybe [you could provide] the information for therapists or support groups. Having access to those resources, maybe places where you could access them for free*.

[Participant 12, 5 years post-MBC diagnosis, completed 10 EMA surveys]

Thus, an EMI that connected patients with peers or provided resources for seeking additional support was widely seen as potentially beneficial.

*Although less common, some participants were not interested in additional resources*. A smaller group of participants, typically those further from MBC diagnosis, was not interested in receiving resources for managing symptoms. As one participant described, “There’s nothing right now that I need addressed. I’m already addressing it. Like I said, I’ve already been around the block a few times, so I’ve already come up with my own way if there is a way” [Participant 27, 5 years post-MBC diagnosis, completed 7 EMA surveys].

## Discussion

### Principal Findings

This study qualitatively explored patient experiences with EMA and their preferences for an EMI to ameliorate QOL. We identified 5 major themes from qualitative interviews: participant engagement, feedback on study design, impact of self-monitoring, responses to data, and recommendations for future interventions. These results highlight that patients with MBC are open to EMA methodologies for data collection and that EMIs can be valuable to this population.

Patients with MBC were engaged and interested in participating in this EMA study. Many patients were motivated by increasing research inclusivity for this population, underscoring the importance to participants of representativeness in research samples. These findings are consistent with other studies, which identified that altruism highly impacts patients’ decisions to enroll in research studies [46,47]. Designing recruitment strategies and study methodology to emphasize inclusivity and the altruistic benefits of the research may facilitate recruitment and retention in research studies [48]. However, we also identified that several patients did not fully understand the study design and purpose of the research. This has important implications for the design and implementation of the informed consent process for future studies to ensure that all participants fully understand study procedures before enrolling. For example, future studies with complex EMA designs might incorporate evidence-based tools for improving informed consent, such as the “teach-back” method [49,50].

Furthermore, participant feedback indicated that a preferred study procedure would have included fewer surveys per day for multiple days in a row, with additional time to complete each survey. Some participants noted that their experiences did not change dramatically over the course of the day, which aligns with and builds upon our previously published quantitative analyses [31,32], demonstrating that time of day of the EMA was not significantly associated with QOL or symptom burden. Taken together, these results can help guide the design of future EMA studies that align with the preferences of the MBC community. Despite their suggestions for improving the EMA schedule, participants generally found the EMA design to be straightforward and easy to understand, similar to other studies using EMA in MBC populations. One such study looked specifically at the feasibility of using EMA for symptom burden and management and demonstrated high adherence and retention [51]. This study extends these findings to demonstrate the feasibility of using EMA to assess physical symptoms, psychological symptoms, positive experiences, and overall QOL among patients with MBC.

One unique result from this study was participant discomfort and confusion discussing libido as relevant to their MBC experience. This finding reflects a broader pattern reported in clinical settings, where the majority of women with breast cancer do not discuss the sexual health effects of cancer with their providers [52]. When conversations about sexual function do occur, they are more frequently initiated by the clinical provider [53]. Participants in this study were also confused about the definition of libido and its relevance as a side effect of many MBC treatments. More effective communication between patients and providers about sexual health is needed to increase patient knowledge [54]. Therefore, future studies should explain the rationale for assessing libido, define the term, and offer an opt-out response option to both increase participant knowledge and limit any potential participant discomfort.

Finally, these results have important implications for future interventions. While patients with MBC are open to EMIs with varying content and formats, more research is needed on the best time to intervene for each person. Intervention timing is an important avenue for future research to explore, as participants in this study varied on when they would have preferred help (ie, in moments of high distress vs the next day). Future research should also further examine patient preferences toward receiving help during moments of high distress to determine feasibility and acceptability of various options (eg, connecting with a peer vs receiving resources). We also observed variability in participant openness toward receiving their data in real time, with some participants feeling positively toward the idea and others negatively. Given this variability in preferences, a just-in-time adaptive intervention (JITAI) may be well suited for this population; JITAIs deliver personalized support in real time, typically using mobile technology, with the aim of providing the right type and amount of support at the right time and place to maximize effectiveness [55]. A JITAI for people with MBC could personalize intervention delivery based on participant and contextual factors, including participant preferences. In terms of the intervention components to be included in a JITAI framework, participants suggested a wide variety of resources, primarily targeted toward patients living with MBC but potentially also engaging family members and support people. These findings build upon previous research, as other studies also document the importance of more MBC-specific support groups to better meet this population’s needs, as well as the lack of formal resources for family members and support people [56]. This study builds on this prior literature and further illuminates current gaps in interventions to improve QOL for patients with MBC.

### Strengths and Limitations

This study is among the first to explore the acceptability of EMA study designs among patients with MBC, a group with significant QOL decrements and few options for tailored supportive care interventions. Our sample included participants who had been living with MBC for varying lengths of time and could therefore contribute unique perspectives on their disease. The qualitative interview design incorporated patients’ EMA data, adding depth and clarity to study findings. We used a robust coding approach involving multiple coders for each transcript and discussion of discrepancies until consensus was reached, which allowed us to mitigate potential individual biases in the coding process.

However, the interpretation of these results must be informed by the study’s limitations. First, interview participants represent a subset of individuals who were eligible for and agreed to participate in an EMA-based observational study of QOL. Thus, results may be subject to selection bias; participants who elected to participate in such a study may be more open to EMA and EMI or may have higher QOL than individuals who declined to participate. Second, responses may be subject to social desirability bias due to lack of anonymity during study interviews. Third, most participants were non-Hispanic White and highly educated. Although representative of patients at National Cancer Institute-designated Comprehensive Cancer Centers [57], it is possible that theoretical saturation was achieved only due to the homogenous nature of the cohort [58]. Relatedly, all participants in this study were patients at academic medical centers; their experience may not reflect the majority of patients with MBC who are seen in community oncology practices [59]. Additional research, including heterogeneous patient populations receiving care in varied settings, would increase the fidelity and usefulness of the findings presented here. Finally, this is a small, qualitative study. Future studies should quantitatively examine the feasibility and acceptability of EMI in a large, representative sample of patients with MBC.

### Conclusions

This qualitative study identified that patients with MBC are willing to use EMA methodologies for data collection on QOL and that EMI could be beneficial for this population. Participants engaged well with the study design, found the EMA framework easy to understand, and would be open to EMIs. A JITAI framework may complement EMA well for this population, with the most frequently suggested interventions including MBC-specific support groups, peer navigation, and resources for family members and support people. Future research will take into consideration this qualitative participant feedback to formulate an updated EMA approach paired with a useful EMI.

## Supplementary material

10.2196/80467 Multimedia Appendix 1 Ecological momentary assessment surveys.

10.2196/80467 Multimedia Appendix 2 Qualitative interview guide.

10.2196/80467 Checklist 1 COREQ (Consolidated Criteria for Reporting Qualitative Research) checklist.

## Abbreviations

EMA: ecological momentary assessment
EMI: ecological momentary intervention
GI: gastrointestinal
JITAI: just-in-time adaptive intervention
MBC: metastatic breast cancer
QOL: quality of life
REDCap: Research Electronic Data Capture
