# Supplementary material for: Evaluation of Experiences With Ecological Momentary Assessment Among Women With Metastatic Breast Cancer: Qualitative Study
Source: JMIR Cancer. 2026 Mar 3;12:e80467. doi: 10.2196/80467 (PMC12977331; doi:10.2196/80467)
Supplement: Multimedia Appendix 1 [file cancer-v12-e80467-s001.pdf]

Please indicate your thoughts regarding each statement by moving the slider along the line.

1. Quality of life is your overall enjoyment of life, including your physical, emotional, and social well-being. How would you rate your quality of life right now?

0 | 100

Worst imaginable | **Perfect**

2. Depression includes feelings of sadness and lack of interest in pleasant activities. What is your level of depression right now?

0 | 100

Not at all depressed | **Extremely depressed**

3. Anxiety includes feelings of tension and worry. What is your level of anxiety right now?

0 | 100

Not at all anxious | **Extremely anxious**

4. What is your level of pain right now?

0 | 100

No pain | **Worst pain imaginable**

5. Fatigue is extreme tiredness and lack of energy. What is your level of fatigue right now?

0 | 100

No fatigue | **Extremely severe fatigue**

6. How is your thinking right now?

0 — 100

Good, my thinking is sharp and quick — **Bad, my thinking is difficult or slow**

7. How is your appetite right now?

0 — 100

Complete lack of appetite — **Normal appetite**

8. Nausea is a feeling of sickness or discomfort in the stomach that may come with an urge to vomit. What is your level of nausea right now?

0 — 100

No nausea — **Extremely severe nausea**

9. Gastrointestinal (GI) distress includes stomach pain, gas, bloating, constipation, and diarrhea. What is your level of GI distress right now?

0 — 100

No GI distress — **Extremely severe GI distress**

10. Libido refers to sexual desire and interest. How is your libido right now?

0 — 100

Complete lack of sexual desire/interest — **Normal sexual desire/interest**

11. How connected to other people do you feel right now?

0 ———————————————————————— 100

Not at all connected | **Extremely connected**

12. How peaceful do you feel right now?

0 ———————————————————————— 100

Not at all peaceful | **Completely at peace**

13. How joyful do you feel right now?

0 ———————————————————————— 100

Not at all joyful | **Extremely joyful**

14. Since [you woke up/the last time you answered questions], did you do anything for self-care?
    - ☐ Yes *(continue to #14a)*
    - ☐ No *(skip to end)*

    14a. **If YES →** What did you do for self-care?

    ____

    ____

    ____
