# Supplementary material for: Evaluation of Experiences With Ecological Momentary Assessment Among Women With Metastatic Breast Cancer: Qualitative Study
Source: JMIR Cancer. 2026 Mar 3;12:e80467. doi: 10.2196/80467 (PMC12977331; doi:10.2196/80467)
Supplement: Multimedia Appendix 2 [file cancer-v12-e80467-s002.pdf]

Interview Guide

Study ID: ________________________
Date/Time: ________________________
Interviewer: ________________________

[TOPIC: Introduction]

Thank you for participating in our study! This interview has two parts. First, I'll be asking you to tell me about your experience participating in our study, and ask for your feedback on how we can improve the study. Then, we'll spend a little time discussing your responses that you shared with us over the course of the study.

[TOPIC: Experiences in the study]

First, I'd like to ask what it was like for you overall to participate in this study. How would you describe your experience?

Potential areas to probe:

- What initially interested you in this study?
- What did you like and/or dislike about participating in this study?
- How easy was it for you to answer the surveys that were sent to you via text message? Did you have any technical difficulties?
- Did any of the questions on the text message surveys make you feel uncomfortable?
- Do you have any feedback for us on how we could improve the study?

[TOPIC: Interest in alternative study designs]

For future studies, we're considering taking a different approach to the text message surveys. Instead of spreading them out over the course of a month, we might ask future participants to complete surveys for multiple days in a row, but for a shorter period of time. What is your initial reaction to this idea?

Potential areas to probe:

- Would you be willing to complete mini-surveys 3 days in a row? What about a full week (that is, 7 days in a row)?
    - Would this approach be easier or harder than our current approach? Why?
    - What barriers or problems would you foresee if we decided to send the mini-surveys multiple days in a row?
    - Would you see any advantages to completing the mini-surveys multiple days in a row?
- What about completing the mini-surveys every other day? Would you prefer that over the current approach (1 day per week)? Would you prefer that over doing them every day?

[TOPIC: Self-monitoring and behavior change]

As part of the study, you were asked to tell us about your experience in the moment. What was it like for you to think about and monitor your experiences?

Potential areas to probe:
- Some people have difficulty identifying their experiences in the moment. How easy or difficult was this part of the study for you?
- Some people become distressed or upset when they're asked to keep track of their experiences (particularly negative experiences, like pain or depression). Was this ever distressing or upsetting for you?
- Some people change their behaviors when they're asked to keep track of their experiences. Did you notice that you changed your behavior while participating in this study?

[TOPIC: EMA data]

These graphs show your responses to the text message surveys. You can see how your responses changed over time. (Briefly explain the graphs, including the vertical axis and the anchors.) Looking at these graphs, what comes to mind for you?

Potential areas to probe:
- Do these graphs seem like an accurate representation of your experiences over the last few weeks? (Ask about any particularly high/low periods on the graph.)
- If you had been shown these graphs in real-time, how do you think you would have responded? Would you have changed anything about your behavior? Why or why not?

[TOPIC: Intervention preferences]

In the future, we hope to use data like this to provide people with suggestions for managing their symptoms. How would you like to receive suggestions for managing your symptoms?

Potential areas to probe:
- When would you like to receive suggestions for managing your symptoms?
- How would you like to receive suggestions for managing your symptoms? Text? Email?
- What kinds of suggestions do you think would have been acceptable/helpful to you in the moment? (i.e., at the time when you were experiencing high symptom burden)

[TOPIC: Additional input]

Thinking about your whole experience participating, is there any other feedback you would like to provide?

[End of interview]

Thank you very much for your honesty and willingness to participate in this discussion. I have learned a lot from talking with you, and our study team really appreciates your input.
