# Supplementary material for: Evaluation of Experiences With Ecological Momentary Assessment Among Women With Metastatic Breast Cancer: Qualitative Study
Source: JMIR Cancer. 2026 Mar 3;12:e80467. doi: 10.2196/80467 (PMC12977331; doi:10.2196/80467)
Supplement: Checklist 1 [file cancer-v12-e80467-s003.pdf]

Consolidated Criteria for Reporting Qualitative Research (COREQ) Checklist

Topic	Item No	Guide Questions/Description	Page No
**Domain 1: Research team and reflexivity**			
Personal characteristics			
Interviewer/facilitator	1	Which author/s conducted the interviews?	3
Credentials	2	What were the researcher's credentials?	1
Occupation	3	What was their occupation at the time of the study?	3
Gender	4	Was the researcher male or female?	3
Experience and training	5	What experience or training did the researcher have?	3
Relationship with participants			
Relationship established	6	Was a relationship established prior to study commencement?	3
Participant knowledge of the interviewer	7	What did the participants know about the researcher? (e.g., personal goals, reasons for doing the research)	3
Interviewer characteristics	8	What characteristics were reported about the interviewer/facilitator? (e.g., bias, assumptions)	3
**Domain 2: Study design**			
Theoretical framework			
Methodological orientation and theory	9	What methodological orientation was stated to underpin the study? (e.g., grounded theory, discourse analysis, ethnography, phenomenology, content analysis)	2
Participant selection			
Sampling	10	How were participants selected? (e.g., purposive, convenience, consecutive, snowball)	2-3
Method of approach	11	How were participants approached? (e.g., face-to-face, telephone, mail, email)	2-3
Sample size	12	How many participants were in the study?	4 and Fig. 2
Non-participation	13	How many refused to participate/dropped out? Reasons?	4 and Fig. 2
Setting			
Setting of data collection	14	Where was the data collected? (e.g., home, clinic, work)	3
Presence of non-participants	15	Was anyone present besides the participants and researchers?	3
Description of sample	16	What are the important characteristics of the sample? (e.g., demographic data, date)	Table 1
Data collection			
Interview guide	17	Were questions, prompts, guides provided by the authors? Was it pilot tested?	Suppl 3
Repeat interviews	18	Were repeat interviews carried out? If yes, how many?	3
Audio/visual recording	19	Did the research use audio/visual recording to collect the data?	3
Field notes	20	Were field notes made during and/or after the interview?	3
Duration	21	What was the duration of the interviews?	3
Data saturation	22	Was data saturation discussed?	3
Transcripts returned	23	Were transcripts returned to participants for comment and/or correction?	3

COREQ Checklist (continued).

**Domain 3: Analysis and findings**			
Data analysis			
Number of data coders	24	How many data coders coded the data?	4
Description of coding tree	25	Did the authors provide a description of the coding tree?	Table 2
Derivation of themes	26	Were themes identified in advance or derived from the data?	4
Software	27	What software was used to manage the data?	4
Participant checking	28	Did participants provide feedback on the findings?	3
Reporting			
Quotations presented	29	Were participant quotations presented to illustrate the themes/findings?<br>Was each quotation identified? (e.g., participant number)	Tables 3-4
Data and findings consistent	30	Was there consistency between the data presented and the findings?	9-13
Clarity of major themes	31	Were major themes clearly presented in the findings?	9-13
Clarity of minor themes	32	Is there a description of diverse cases or discussion of minor themes?	9-13
